# The Mind–Matter Dichotomy: A Persistent Challenge for Neuroscientific and Philosophical Theories

**DOI:** 10.1111/ejn.70143

**Published:** 2025-05-19

**Authors:** Wolf Singer

**Affiliations:** ^1^ Ernst Strüngmann Institute for Neuroscience in Cooperation with Max Planck Society, Max Planck Institute for Brain Research, and Frankfurt Institute for Advanced Studies Frankfurt am Main Germany

**Keywords:** cognitive neuroscience, consciousness, cultural evolution, mind–body problem, neuropsychology, ontological dualism, perception, philosophy of mind, qualia, self‐model

## Abstract

Several areas of cognitive neuroscience tackle traditional philosophical questions. Among the range of problems, two closely related issues will be addressed in more detail from both neurobiological and philosophical perspectives: the relationship between mind and matter and the nature of perception. Neuropsychological and neurophysiological studies are reviewed that examine the connection between neuronal processes and consciousness. The most prominent theories on the neuronal correlates of consciousness (NCC) are then compared with philosophical attempts to address the epistemic gap between the material processes in the brain and mental phenomena. Before exploring whether neurobiological discoveries can help resolve philosophical problems, the epistemic challenges are discussed, stemming from the fact that perceptions are shaped by the brain's functional architecture. It is suggested that the ‘hard problem of consciousness’—the challenge of explaining how the qualia of subjective experience can arise from neuronal processes—can be alleviated if two conditions are met: first, that perception depends on priors and, second, that some of these priors are formed through interactions with the immaterial realities of cultural concepts. Although this approach offers a coherent naturalistic explanation for the emergence of mental phenomena, it does not resolve the cognitive dissonance between our intuitions and scientific evidence regarding the relationship between matter and mind.

## Graphical abstract

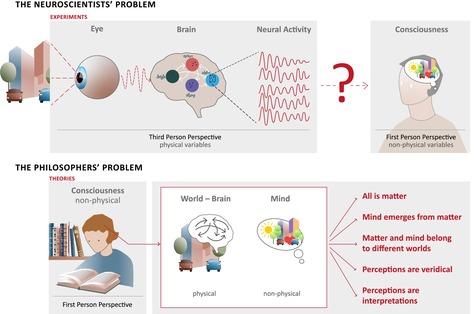

## 1 Similarities Between Science and Philosophy

Novalis, a writer and philosopher of the early German Romanticism, once stated: ‘Im Grunde ist jeder Mensch ein Philosoph’, meaning that basically, every human is a philosopher. And Jakob Bronoswski, a British mathematician and historian of science (1908–1974), concluded ‘All humans are scientists’. If one considers these statements as valid, they imply that all scientists are philosophers and, vice versa, all philosophers are scientists.

Indeed, the goals pursued by these two disciplines are strikingly similar, as are the tools used to attain them. Philosophical and scientific inquiries both aim at fundamental questions regarding the nature of reality and our existence. Concrete examples of the overlap between scientific topics and philosophical questions include in physics, the nature of matter, time and space; in biology—encompassing biochemistry, molecular biology and evolutionary biology—the nature of life and the emergence of new qualities through self‐organization; and in neuroscience, virtually all phenomena related to higher cognitive functions, such as the foundations of perception, knowledge acquisition, memory, emotions, decision making, volition, reasoning, value assignment, language, aesthetic and moral judgements, distinctions between self and other, theory of mind and, last but not least, the relationship between matter, mind and consciousness. The phenomena to be explained (explananda) in philosophical inquiry cover a much wider range than those in the natural sciences, as they also include phenomena arising from social interactions that initiated cultural evolution. These explananda encompass the nature of knowledge, the origin of values and meaning, the foundations of logic and reasoning, the concepts of morality, justice, truth, freedom, culpability, beauty and many others, including religions and belief systems.

A remarkable overlap also exists between philosophy and science, concerning the methods of inquiry utilized by both disciplines. Ultimately, both derive their explanations from either observations or a body of previously formulated concepts, theories and hypotheses. Both employ critical reasoning, logical deduction and induction, intuition and aesthetic criteria to gain novel insights. They also use similar strategies to support the validity of their conclusions: thought experiments in philosophy, control experiments in the sciences and debates aimed at uncovering contradicting or incompatible evidence in both fields. Although mathematics is indispensable in the sciences, philosophers have also used mathematics since antiquity to formulate their concepts or to leverage the rigour of mathematical reasoning as a criterion for the consistency of philosophical theories. Prominent examples include Plato, Descartes, Leibniz, Kant, Russell, Gödel, Wittgenstein, Whitehead and Carnap, as well as the phenomenologist Husserl, who began his career as a mathematician. Just as the explanations in philosophy extend beyond the natural sciences, the tools of the natural sciences also surpass the toolkit of philosophers. This primarily pertains to the observational aspect of scientific work, the enhancement of natural senses through instruments, the validation of observations using statistical methods, the analysis of results with advanced mathematical tools and the modelling of systems whose complexity and nonlinear dynamics challenge mathematical analysis through computer simulations.

Another parallel between science and philosophy is the increasing specialization. Over the last five centuries, beginning with the Renaissance that initiated the programme of enlightenment, the natural sciences have undergone unprecedented differentiation into various disciplines. The same trend has occurred in philosophy. This may partly result from the diverse scientific discoveries that required adaptation within philosophical theories. However, once philosophy established itself as an independent academic discipline, its internal dynamics likely fostered specialization as well. The major branches of philosophy—metaphysics, epistemology, ethics, logic and aesthetics—have been complemented by subfields that address specific scientific issues more directly. Under the umbrella of the philosophy of science, subdisciplines now focus, among others, on physics (the nature of matter, space and time), chemistry (the origin of life), medicine (ethics of interventions), biology (evolution and emergence of new qualities) and neuroscience (the nature of perception, memory, the mind and consciousness). As neurobiological discoveries, particularly in cognitive neuroscience, are especially pertinent to many enduring philosophical questions, neurophilosophy and the philosophy of mind have rapidly emerged as growing subfields within philosophy.

The high degree of specialization in the sciences, driven mainly by the diversification of methods of observation, has led to communication problems between disciplines. Even within specific fields—neuroscience being a prominent example—the level of specialization has become so high that different descriptive systems must be employed to capture the structural and functional aspects of nervous systems at various levels. This phenomenon is reflected in the massive proliferation of specialized journals. Experts in molecular neurobiology often find it challenging to review a paper presenting findings in cognitive or computational neuroscience, and vice versa. Thus, the field becomes lost in translation.

I cannot judge whether the specialization in philosophy has led to similar problems or whether philosophers of religion and philosophers of the mind communicate with one another and understand their respective jargon. From a distant perspective, it seems, however, that the methods of investigation used in the different subfields of philosophy are more similar than those in the various scientific fields. Thus, if scientists can successfully translate their findings into the language used by philosophers, philosophy might help to integrate some of the diverse observations from the sciences into overarching concepts, thereby contributing to bridging gaps between the unique descriptive systems of scientific subdisciplines. If this assumption is true, synergistic interactions with philosophy could be fostered if scientists familiarize themselves sufficiently with philosophical toolkits to express their observations in terms recognizable to philosophers, particularly those focused on their respective disciplines.

## 2 Topics in the Focus of Neuroscience and Philosophy

Which domains do the neurosciences directly address regarding traditional philosophical questions, and in what ways could philosophical tools be particularly relevant to the neurosciences? Among the array of problems, three will be discussed in more detail: the nature of perception, the neuronal correlate of consciousness (NCC) and the relationship between mind and matter. These issues are closely interconnected, as they all explore the connection between neuronal mechanisms described from a third‐person perspective and subjective phenomena that are accessible only from a first‐person perspective.

Both cognitive neuroscience and epistemology explore the extent to which our perceptions are veridical and the criteria for veridicality. The question is whether our perceptions are constrained by the functional organization of our brains and, if so, which aspects are affected. If our perceptions are influenced by the organization of our brains, what implications does that have for our model of the world and our self‐model—the way we perceive ourselves? Furthermore, if these constraints vary between individuals due to genetic differences and epigenetic modifications, our perceptions would be idiosyncratic, raising additional epistemic questions.

Theories of perception and the NCC address the epistemic challenge of the gap between the neuronal mechanisms that produce perceptions and the qualities of subjective experience. Due to the fundamental nature of this challenge, I will prioritize the discussion of neurobiological and philosophical theories of consciousness. This phenomenon carries a multitude of connotations in both the neurosciences and various philosophical traditions, raising the question of whether ‘consciousness’ serves as an umbrella term for a cluster of phenomena, with numerous theories (see below) tackling different but related aspects of consciousness. For me, it is challenging to formulate a canonical definition. What St. Augustine of Hippo (1961) remarked when asked about the nature of time (Confessions, book 11, chapter 14) appears to apply to consciousness as well: ‘What then is time? If no one asks me, I know what it is. If I wish to explain it to him who asks, I do not know’.

## 3 A Brief History of Scientific Explorations of Consciousness

Despite or because consciousness is such an elusive explanandum, it has been the focus of philosophical inquiry since antiquity and is today at the core of several philosophical disciplines, particularly the rapidly expanding philosophy of mind. Key figures include David Chalmers (1996), Daniel Dennett (1991), Thomas Metzinger (2003, 2009), Patricia Churchland (2002, 2013), Ned Block (1995) and Anil Seth (2021).

However, it was only in the 19th century that phenomena manifesting solely from the subjective, first‐person perspective—such as perception, awareness and consciousness—were included in the agenda of scientific investigations. Scientists such as Mach, Wundt and Helmholtz (reviewed by Farell 2014) set out to study the physical laws underlying psychological phenomena, founding psychology and, more specifically, ‘psychophysics’ as new scientific disciplines. William James, in his seminal book ‘The Principles of Psychology’ (1890), discussed the ‘stream of consciousness’, emphasizing the continuity and connectedness of the contents appearing in consciousness. Neuroanatomists and neurologists like Broca and Wernicke, to name just a few, studied the correlations between cognitive deficits and focal brain lesions, establishing close relationships between the sites of brain lesions and disturbances related to conscious processing. These investigations are at the roots of a new discipline now called neuropsychology. This led to systematic investigations of the relations between brain processes and consciousness. Here is a selection of groundbreaking research in this domain: The seminal studies of Moruzzi and Magoun (1949) from Pisa and Jouvet (1999) from Lyon revealed the dependence of consciousness on brain structures controlling arousal; Penfield's stimulation experiments established direct relationships between neuronal activity in circumscribed brain regions and the contents of conscious experience (Penfield and Jasper 1954); Scoville and Milner (1957), in their studies of the famous case HM—a patient who had the temporal lobes, including the hippocampi, removed bilaterally due to intractable epilepsy—discovered that these structures were required for conscious access to past experiences; Gazzaniga and Sperry (1967) and Gazzaniga et al. (1967), in their puzzling studies with split‐brain patients, demonstrated close relations between hemispheric dominance and the verbal reportability of conscious experience, thereby sharpening the distinction between conscious and subconscious processing; two decades later, Weiskrantz et al. (1974), studying blindsight in patients suffering from lesions of the early visual cortex, provided detailed insights into the neuronal substrates supporting conscious and subconscious processing, respectively. Similar progress was achieved in identifying the neuronal underpinnings of other subjective phenomena such as drives and emotions (Schultz 2006; Schultz et al. 2021; Damasio 2005; LeDoux 2012).

Despite convincing demonstrations of a close relationship between conscious processing and neuronal processes, along with abundant evidence that drugs influencing brain states lead to altered states of consciousness (see, e.g., Stoll 1947; Doblin and Burge 2014), as well as clinical observations of strong connections between electrophysiologically confirmed brain states and levels of consciousness, the systematic search for the neuronal underpinnings of consciousness has remained surprisingly sparse. Crick and Koch (1990) published a provocative paper in which they declared the search for the neuronal correlates of consciousness (NCC) a legitimate scientific agenda. Since then, the investigation into the neuronal underpinnings of consciousness has gained momentum. Psychophysical, neurophysiological and theoretical studies have proliferated with the goal of identifying the NCC (for reviews of the extensive literature, see Velmans and Schneider 2007, 2019; Zelazo et al. 2007; Seth and Bayne 2022).

However, difficulties with defining consciousness and establishing criteria for conscious processing persist. In human studies, the verbal reportability of stimuli is typically regarded as evidence of conscious processing. Consequently, most imaging and electrophysiological studies investigating the Neural Correlates of Consciousness (NCC) depend on the subtraction method. Brain activity triggered by subliminal, nonperceived and nonreportable stimuli is subtracted from the activity induced by the same stimuli when they are reported as perceived, and the remaining activity is considered to reflect the NCC. However, this widely applied approach is burdened with methodological and conceptual issues (see, e.g., Aru et al. 2012). This is particularly problematic in animal experiments, where only operant responses are available, making it challenging to distinguish between conscious and subconscious processing.

Nevertheless, all the most influential theories assume that specific neuronal circuits or dynamic states must be engaged when subconscious processing transitions to conscious processing. In mammals, preferred candidates are recurrent cortical or thalamocortical circuits (see below). In nonmammalian vertebrates, such as birds, which lack the characteristic six‐layered cortex of mammals, structures thought to be responsible for conscious processing exhibit connectivity patterns—abundant recurrency—that resemble those of the mammalian neocortex (Güntürkün 2005; Nieder et al. 2020).

## 4 Current Theories on the NCC

Because Crick and Koch (1990) proposed that consciousness can be studied scientifically like any other cognitive function, empirical studies exploring the neuronal correlates of consciousness have increased, along with the theories developed by neuroscientists. In this context, comprehensive reviews and encyclopaedias are already available (Seth and Bayne 2022; Velmans and Schneider 2019). Therefore, I will focus only on a subset of the theories that are suitable for establishing connections to philosophical positions.

Motivated by the lack of evidence for a specific brain area identifiable as the seat of consciousness, Baars (1988, 2007) proposed in the 1980s that there should be a ‘global workspace’ for conscious processing. This global workspace theory (GWT) was further developed by Dehaene and Changeux (2011), who suggested that this workspace comprises a widely distributed network of neurons located in the supragranular layers of the cerebral cortex. The idea is that this network is ‘ignited’ and engages in cooperative interactions when the threshold separating subconscious from conscious processing is crossed. In this concept, it is the enhanced activity of a subnetwork that supports conscious processing.

Somewhat related is the theory proposed by Varela et al. (2001) (Singer 2007, 2017; Melloni et al. 2007; Fries et al. 1997; Engel and Singer 2001). These authors suggested that the correlate of conscious processing is a specific dynamic state of cortical networks characterized by enhanced coherence or synchrony. The conceptual foundation of this theory is the ‘Binding by Synchrony’ hypothesis (Singer 1999). This hypothesis posits that semantic relationships among the contents signalled by neuronal responses are encoded through the temporal relations between responses. Such responses are considered oscillatory (Singer and Gray 1995; Gray and Singer 1989; Gray et al. 1989), allowing the definition of temporal relations in phase space through coherence and synchrony. All components of a conscious experience are interconnected, a phenomenon referred to as the ‘unity of consciousness’ (see Immanuel Kant 1781 in Guyer and Wood 1998; James 1890). Thus, the signature of conscious states is thought to be enhanced binding through temporal coherence among the responses of distributed ensembles of cortical neurons that encode the actual contents of conscious experience. As these contents and their relationships constantly evolve, dynamic and flexible binding operations are necessary. It has been suggested that such flexible binding could be achieved by representing component features of contents through the discharge amplitude of neurons and the varying relationships between the components via the phase relations among oscillating responses (Singer 2021). Therefore, the seamless unity of contents in the continuous stream of consciousness would not be ensured by anatomical convergence. Instead, the representation of these contents would consist of assemblies of widely distributed neurons, with their interrelatedness expressed through synchrony or other signatures of coherence in phase space. Sherrington (1940) anticipated this interpretation when he noted in his book ‘Man on His Nature’ that ‘the unity of consciousness is due to unity in time rather than in space’.

Penrose and Gardner (1989) suggested that the holistic nature of consciousness, alongside its dynamics and the inability to link its source to specific brain structures, cannot be explained solely within the framework of classical physics. Therefore, they speculated that phenomena observed in quantum physics, such as the superposition of wave functions, their collapse upon measurement and the entanglement of states, could offer potential solutions (Penrose and Gardner 1989). Sceptics contended that the brain's warm and wet environment was unsuitable for supporting quantum states like entanglement. However, Hameroff and Marcer (1998) explored this idea further and proposed that the network of microtubules possesses properties capable of sustaining quantum effects. Although this latter hypothesis has garnered limited support, recent studies of networks of coupled oscillators reveal that their dynamics exhibit certain similarities to those of quantum systems (Babbush et al. 2023). Recurrent networks with nodes arranged as oscillators demonstrate nonlinear dynamics characterized by travelling waves and complex interference patterns akin to the superposition of wave functions, which can be utilized for highly parallel computations. Simulation studies of such networks have shown that they replicate numerous characteristic features of cortical dynamics, facilitating a holistic representation of information and efficient parallel computations in phase space (see Effenberger et al. 2023, 2025 for details and further literature). Thus, the arguments that led Penrose and Gardner (1989) to propose quantum effects as the foundation of consciousness remain valid, yet the suggested computational principles can also be realized in conventional recurrent neuronal networks, provided that their nodes have the propensity to oscillate, which has been shown to be the case (Gray and Singer 1989).

Another theory regarding the NCC, the ‘Information Integration Theory’ (ITT), was introduced by Tononi (2004) and is based on an information‐theoretical approach. Tononi proposes a circuit model as a substrate for conscious processing, enabling maximal integration of information, and introduces a mathematical method to quantify the amount of integrated information (phi). The theory posits that the level of consciousness increases with phi. This measure can be derived from both anatomical and functional connectivity. In the latter scenario, phi has been shown to increase with the radius of activity spreading across the cortex following focal stimulation, and this radius is positively correlated with the level of arousal (Tononi and Massimini 2007).

Finally, several authors have proposed a relationship between the NCC and processes related to predictive coding (Friston 2010; Friston et al. 2020; Seth 2021; Seth and Bayne 2022; Hohwy 2013). These authors suggest that brain states associated with a strong match between sensory evidence and priors—the internal model of the world—are linked to conditions in which processed contents are accessible to conscious experience. Friston (2010) equates these conditions with those of minimal free energy, facilitating mathematical definitions and some quantification. Seth (2021) describes the contents of conscious experience as the ‘brain's best guess’, an intuitive expression of strong matches between sensory evidence and the internal model of the world. States that represent strong matches, in turn, correspond to states of minimal free energy.

Currently, the protagonists of these theories are making a concerted effort to collaborate on the design of adversarial experiments and test critical predictions (Cogitate Consortium et al. 2023). It is important to note that most, if not all, of the experimental evidence underlying these theories is, so far, only correlative. This is because gain and loss of function interventions in complex interconnected systems tend to interfere with more than just the target variable.

Another branch of consciousness research examines altered states of consciousness associated with clinical conditions. These studies are not only relevant for identifying the NCC but are essential for informing ethical decisions regarding the treatment of comatose patients. Here, the primary focus is on establishing connections between brain functions and levels of consciousness, which range from minimal states of consciousness to full awareness (see Laureys et al. 2015; Sanz et al. 2021). These studies uncover strong correlations between levels of consciousness and the functional integrity of distinct brain structures, as well as the dynamics of global brain states.

Strong correlations between brain activity and subjective feelings are evident in altered states of consciousness associated with epilepsies and hallucinogenic drugs. Focal epilepsies in certain brain structures, such as the anterior insula and the cingulate gyrus, can lead to blissful experiences that closely resemble feelings of enlightenment and religious awakenings (Picard 2023; Devinsky and Lai 2008; Timmermann et al. 2023). Similar states are reported to be induced by hallucinogenic drugs and rituals involving sleep deprivation, hyperventilation and rhythmic dancing (Cardeña and Winkelman 2011).

Although these searches for the NCC focus on the brain, cognitive neuroscientists are increasingly interested in the intimate reciprocal interactions between the brain and the body and, by extension, between the organism and the environment. The recent concepts of ‘enaction’ and ‘embodiment’ represent the notion that investigations and interpretations of brain functions, including consciousness, must adopt a more holistic strategy. Noë (2004) emphasizes that perception arises through ‘sensorimotor contingencies’, linking perception and action in a nonrepresentational way (Noë 2004; Varela et al. 1991; Thompson 2007; Gallagher 2005; Shapiro 2011).

## 5 Philosophical Theories on the Nature of Consciousness

All neurobiological attempts to identify the NCC assume that consciousness arises from neuronal processes, largely sidestepping the questions central to philosophical considerations regarding the nature of consciousness. Experimental approaches in search of the NCC typically investigate the neuronal underpinnings of a specific cognitive function deemed characteristic of conscious processing. In contrast, philosophical theories mainly explore the epistemic questions concerning the relationship between the material processes in the brain and organism on one side, and the subjective, immaterial quality of consciousness and its contents on the other.

Philosophical theories about the nature of consciousness vary significantly in their perspectives on the relationship between the mind and the brain. Given the profound implications these concepts hold for our self‐model, it would be advantageous if neurobiology could help validate one theory over another. However, before exploring this possibility, it is essential to briefly review some of the most prominent theories. This discussion will reveal that some theories cannot be validated or falsified using scientific methods. As I am not a philosopher, I apologize in advance for any omissions, inaccuracies in terminology and a certain degree of naivety. While writing this text, I realized how challenging it is to navigate unfamiliar descriptive systems.

The philosophical theories addressing the mind–matter problem encompass the entire spectrum of conceivable relationships between the brain and consciousness or, more generally, between mind and matter. These theories range from materialism and physicalism to various forms of emergentism, idealism, panpsychism and ontological dualism. The radical theories of materialism or physicalism posit that everything is matter and that the mind is ultimately reducible to neuronal interactions (see, e.g., Kim 1998, 2018). At the other extreme, idealism argues that reality is fundamentally spiritual, suggesting that consciousness is primordial whereas matter is secondary (see, e.g., Bradley 1893). This perspective is upheld not only by idealist philosophical schools but also lies at the heart of religious beliefs, Buddhist philosophies and many other Western and Eastern spiritual traditions (for a discussion, see Ricard and Singer 2017). A related viewpoint is taken by neutral monism (see, e.g., Russell 1921), which posits that both mind and matter depend on a common primordial substance that is neither physical nor spiritual. In this case, the mind and matter—specifically, mental processes and the physical brain—are merely different manifestations of this substance. Closer relationships between the brain and mind are discussed in theories associated with emergentism (for a comprehensive summary, see Kriegel 2020). These theories propose that consciousness, referring to the mental or spiritual dimension, emerges from neuronal interactions but is not identical to brain processes. Some of these theories draw an analogy to the emergence of new qualities in self‐organizing complex systems that exhibit nonlinear dynamics and evolve far from thermodynamic equilibrium. A frequently cited example is the formation of geometric patterns in autocatalytic diffusion–reaction systems (Nicolis and Prigogine 1977; Haken 1977; Zhabotinsky and Belousov 1964).

Several variants of emergentist theories have been proposed. All of them share the idea that new qualities cannot be solely deduced from the properties of the components of a complex system but must also take into account the dynamics of their interactions. However, the various theories differ in the extent to which mental phenomena rely on the physical processes underlying them.

Weak Emergentism holds that the emergent property can ultimately be understood through a comprehensive analysis of the underlying low‐level processes, whereas strong Emergentism challenges whether emergent phenomena can be fully explained by these foundational mechanisms. Additionally, weak Emergentism suggests that once consciousness manifests, it can influence processes at the lower level. Thus, it takes a clear stance in the debate regarding whether consciousness is merely an epiphenomenon or has a functional role in brain processes (Chalmers 1996). Although these emergentist theories closely align with neurobiological theories on the NCC (see above), emergent dualism and nonreductive physicalism grant greater independence to the mental dimension. These latter theories assert that consciousness emerges from brain processes, but once it manifests, it is seen as ontologically distinct from those processes. It is nonphysical and necessitates descriptive systems that differ from those used for the underlying physical neuronal mechanisms. Feelings arise from neuronal activity, correlate with neuronal activity in specific structures but cannot be reduced to neuronal activity.

Although the emergentist theories discussed above still suggest a close, sometimes even causal, relationships between neuronal processes and mental phenomena, supervenience theories posit correlative relationships. They argue that conscious states are closely linked to brain states but cannot be reduced to neuronal processes. The question of how these correlations arise remains unanswered. Is there a hidden, yet‐to‐be‐identified variable that coordinates neuronal and mental processes?

Efforts to resolve this conundrum are articulated in panpsychist theories (see, e.g., Goff 2017), some of which incorporate elements of emergentism. One assumption is that consciousness is a fundamental property of matter whereas the complexity of manifested consciousness depends on the complexity of the relevant material substrate. For human consciousness to manifest, a complex system such as the human brain is necessary. This line of reasoning can also be extended beyond the brain. One perspective suggests that human consciousness requires a human brain within a human body to express itself. This concept of embodiment is similarly recognized, to varying degrees, by other emergentist theories. As previously noted, the neuroscientific community increasingly recognizes that analysing brain functions necessitates considering the intimate reciprocal interactions with the surrounding body and, by extension, with the environment in which the organism evolves.

## 6 Questions Related to Philosophical Theories

All philosophical positions regarding the mind–matter relationship are fraught with problems that have, in turn, sparked interesting and controversial debates. Within the framework of radical physicalism, as well as Chalmers' property dualism, consciousness can be seen as an epiphenomenon without any function (Chalmers 1996, 2017). Consciousness merely serves as a scratchpad for ongoing neuronal processes and does not influence the organism's behaviour. To address the persistent issue of why material and mental processes are experienced as distinct, proponents of radical physicalism often adopt a panpsychist perspective, redefining matter as having some spiritual qualities.

Theories related to emergentism must clarify the mechanisms by which material processes can give rise to phenomena such as thoughts, reasoning, emotions, the qualia of perception, awareness and, ultimately, self‐awareness and consciousness. The question remains as to how the immaterial quality of the mind can emerge from the brain's material substrate. This challenge persists, even as neuroscience identifies the NCC, the neuronal correlates of conscious processing. This is why this apparent phase transition from material processes to the immaterial, subjective contents of consciousness is referred to as the ‘hard problem’ of consciousness research (Chalmers 1995). The final chapter of this contribution briefly discusses this conundrum.

Reverse problems must be addressed by idealistic theories, particularly the extreme forms of substance or ontological dualism. These theories confront the question of how mental processes such as will, decision‐making, moral judgements and arguments can be translated into structured neuronal activity, which is ultimately necessary to execute the mind's intentions. This central question in dualist theories is discussed in the philosophy of mind as the problem of ‘mental causation’. It troubled the late Sir John Eccles when he adopted a dualist stance and proposed that a premotor cortical area functions as the ‘liaison brain’ (Eccles 1994), the location in the brain where mind and matter interact. He interpreted the evidence that a volitional motor act is preceded by a ‘Bereitschaftspotential’ (the readiness potential), an increasing negativity in direct current recordings first described by Kornhuber and Deecke (1965), as an indication of an interaction between mind and brain. However, this raises the second question of how an immaterial entity can interact with matter without exchanging energy. Given the equivalence of energy and matter, the mind should be free of energy. If it possesses the energy to influence neuronal processes, it must be regarded as a form of matter (for a review and references, see, e.g., Popovici 2018). Eccles proposed as a solution the exploitation of quantum effects, with the mind influencing only the probabilities of neuronal transmission (Beck and Eccles 1992). To the best of my knowledge, there are currently no plausible mechanistic explanations for mental causation, at least not within the current framework of the natural sciences.

## 7 Consequences for Models of the World and Ourselves

Our self‐model and, consequently, our judgements on societal issues depend on the philosophical position we adopt in the mind–matter discourse. Accordingly, answers to important ethical questions vary. Adherents of idealist and dualist positions argue that our will is free and not constrained by the deterministic mechanics of our brain. Advocates of materialism and most emergentist positions conclude that our decisions arise from complex neuronal processes and are, therefore, constrained by deterministic neuronal interactions. Dualists grapple with the issue of mental causation. Emergentists must explain why we perceive ourselves as free to decide and act according to our will. Ultimately, they must address how a seemingly autonomous self can constitute itself within a distributed, self‐organizing neuronal system such as the brain.

Having evidence‐based arguments that support one of the hypotheses about the mind–matter relationship would clearly be beneficial. Concepts like guilt, responsibility, punishment and education are at stake. Some may even argue that certain materialist theories challenge human dignity. As cognitive neuroscience investigates the brain mechanisms thought to underlie perception and consciousness, the question emerges: Do neuroscientific insights favour one theory over the other regarding the mind–matter relationship?

## 8 Epistemic Considerations

Before addressing the question of whether neuroscience can provide evidence for or against certain theories regarding the mind–matter relationship, some epistemic considerations need to be discussed—questions that lie at the core of both epistemology and cognitive neuroscience. I will refrain from conducting an in‐depth review of the numerous philosophical theories on the nature of perception that offer various interpretations of the relationship between the perceiving subject and the external world. These theories range from ‘direct realism’ (Bonjour 2004) to ‘phenomenology’ (de Santis et al. 2020). The former assumes that we perceive reality as it is, whereas the latter emphasizes subjective experience and the first‐person perspective instead of making inferences about the nature of reality. Positioned somewhere in between is ‘constructivism’ (Varela et al. 1991), which posits that perceptions, and more broadly, perceived reality, result from interpretations based on prior knowledge. This knowledge is thought to reside in the functional architecture of the brain, which is largely determined by genes and refined through lifelong epigenetic modifications. Hence, prior knowledge, also referred to as priors, comprises information about the structure of the world acquired through both evolutionary adaptation and lifelong learning.

Neurobiological evidence underscores the idiosyncratic nature of perception resulting from the evolutionary adaptation of sensory systems to the ecological niches in which organisms evolved. Due to the specialization of sense organs that varies across species, only a limited range of environmental signals is encoded and made available for further processing. The algorithms by which these sparse signals are transformed to support perception and action are determined by the structural and functional architecture of the respective processing streams. These architectures are also adapted to the specific needs of the organisms, enabling them to cope with the challenges posed by particular environmental conditions. Genetic instructions dictate the basic anatomical layout of these processing architectures, whereas experience‐dependent pruning of connections refines these innate structures, with lifelong learning continuously modifying the strength of these connections. The fundamental layout of this circuitry establishes the computational algorithms through which relevant sensory signals are processed. Thus, the knowledge acquired through evolution regarding significant features of the world and effective strategies to extract and evaluate these features is already embedded in the basic architecture of the sensory systems. This knowledge is further enhanced by experience‐dependent pruning of connectivity during development. Such processes, combined with lifelong learning, instil knowledge about statistical regularities and contingencies of the encountered environment within the functional architecture of sensory systems (for review see Singer 2018, 1995). Consequently, sensory signals are processed by neuronal networks whose functional architecture contains substantial knowledge about the world. Psychophysical and neurophysiological evidence (for review, see Singer 2021) indicates that this knowledge plays a crucial role in shaping our perceptions. What and how we perceive depend on the outcomes of matching operations between sensory evidence and stored knowledge—a process known as ‘predictive coding’ (Rao and Ballard 1999). Furthermore, the sensory signals that ultimately lead to perceptions are actively selected by attentional mechanisms influenced not only by the salience of the signals but also by volition and subconscious factors reflecting the actual needs of the organism. Thus, perceiving is an active, constructive process in which the brain takes the initiative. This idea has led to the concept of ‘enactive cognition’ (Noë 2004; Varela et al. 1991) and aligns with arguments proposed by various philosophical theories, including extreme constructivism (von Glasersfeld 1984; von Foerster 2003), ecological approaches to cognition (Hutchins 2010) and critical realism (Bhaskar 2008). Even phenomenology, at least concerning the subjective aspect of perceptions, is compatible with the notion that perception is an active, constructive interpretative process.

The fact that the internal model shaping perceptions reflects knowledge about the world acquired during both evolution and ontogeny suggests that our perceptions are somehow connected to reality. However, this connection applies only to the mesoscopic dimension of reality in which life evolved and to which our sensory systems have adapted. Our intuitions regarding the nature of matter, time, space and causality are shaped by our experiences with this mesoscopic aspect of the world. Nevertheless, scientific discoveries continually compel us to expand this model of the world with concepts that contradict our intuitions, and there is no reason to think that this process will cease. As a result, we find ourselves in an epistemic circle. Neurobiological research indicates that our cognitive systems are adapted to a very narrow segment of reality, which constrains what we can perceive. Whether these limitations of our cognitive functions also constrain what we can imagine, and potentially how we reason, is a fascinating question. My intuition is that this should be the case. Despite the possibility of being caught in an epistemic circle, we cannot but try to integrate into coherent concepts what is currently regarded as science‐based evidence, remaining fully aware of the preliminary nature of our conclusions.

## 9 The Hard Problem of Consciousness

Cognitive neuroscientists cannot avoid taking a position in the mind–matter debate; they must address the hard problem of consciousness. However, the epistemic caveats mentioned above, along with others I may have overlooked, should be considered. Given the wealth of neurobiological evidence regarding the close relationship between neuronal processes and mental phenomena, neuroscience appears to support philosophical theories that assume a causal relationship between the brain and the mind. The continuity of evolutionary processes aligns with this perspective. There are good reasons to assume that animals can also reason or experience emotions and sensations at a level comparable to conscious processing (Panksepp 2005; Bayne et al. 2024; Nieder et al. 2020). They possess a similar mechanism as our brains for controlling arousal and attention, gating access to working and episodic memory and integrating multimodal information. Behavioural studies reveal that the sophistication of cognitive functions varies among species and is positively correlated with brain differentiation (Mitchell 2016). A similar relationship exists for the developing human brain. Cognitive abilities related to conscious processing become increasingly elaborate as the brain matures (Crotty et al. 2023), and we readily attribute consciousness to babies even before they acquire language. Thus, verbal reportability of the contents of conscious experience is apparently not a sine qua non for conscious information processing. Otherwise, Thomas Nagel's famous question, ‘What Is It Like to Be a Bat?’ (Nagel 1974), would be ill‐posed.

These considerations raise the question of why the hard problem is regarded by some as such an insurmountable conundrum. Granting animals awareness of their perceptions and inner states does not seem to pose an epistemic problem in itself. It is merely one of many cognitive abilities that can be identified from a third‐person perspective. By contrast, it seems that we humans have a problem with the subjective contents of conscious experience, the qualia, that are accessible only from a first‐person perspective. What experiences lead humans to believe that processes in our minds are disjunct from and unconstrained by underlying neuronal processes? Is the problem a consequence of our ability to use a symbolic communication system that allows us to distinguish material from immaterial realities and to label these realities as belonging to different ontological categories? An extensive discussion of the possibility that the connotations humans associate with consciousness are grounded in the development of a symbolic communication system is to be found in the book of M. Donald, ‘A Mind so Rare, the Evolution of Human Consciousness’ (2002). In the following, I shall attempt to respond to the questions arising from the mind–matter dichotomy, keeping in mind the epistemic caveats discussed above.

The most likely reason we perceive mental processes as independent of neuronal processes is that our brains are opaque to us (Metzinger 2003). We are unaware of the neuronal interactions that underlie our perceptions, deliberations, feelings, decisions and actions. Instead, we only notice the outcomes, not the machinery that produced them. Thus, an agent is postulated to satisfy the deep‐seated belief that all phenomena have causes and to fulfil the desire to identify and name these causes. In many cultures, the agent deemed responsible for mental phenomena is thought to possess qualities that differ from those of the material world but is still assumed to be real. This raises the question of how this conceptual divide between material and immaterial reality could have emerged, which appears to be at the core of the hard problem of consciousness.

## 10 Cultural Evolution as Cause of the Hard Problem?

Since the dawn of culture, humans have experienced phenomena, primarily resulting from social interactions, that possess qualities distinct from those of the material world. These phenomena are perceived as real, even though they cannot be grasped by the senses attuned to the physical realm. Examples include character traits such as generosity, greed and compassion and, at a more abstract level, the contents of shared beliefs and social concepts such as justice, fairness and freedom. As humans possess language—a communication system that employs symbols—these ‘immaterial’ phenomena can be named and treated linguistically just like concrete objects. They become integrated into our model of the world and enhance this model by introducing an immaterial dimension. Consequently, humans are well acquainted with the existence of an immaterial dimension populated by ‘objects’ that are potent, some endowed with agency and that influence behaviour to a similar extent as conditions of the material world. Thus, they are recognized as an integral part of reality.

The question is whether the hard problem of consciousness could be mitigated if it were considered not only from a neurobiological or philosophical perspective but also in the broader context of cultural evolution.

As discussed above, our perceptions are strongly shaped by priors, most of which are covert, representing implicit knowledge about the world. Consequently, we remain unaware of how they shape our perception and must take for granted what we perceive.

Priors are likely acquired not only through interactions with the material world but also through interactions with cultural realities. If this is the case, our perceptions would also be shaped by priors provided by our cultural embedding. It appears natural, then, that we perceive immaterial cultural realities as given, take them for granted and integrate them into our world model—a model that captures both the material and immaterial aspects of the world and assumes coexistence of the two dimensions. It is the covert nature of priors that makes us humans experience the immaterial dimension of the world as equally constitutive and primordial as its material dimension.

If these cultural priors also shape our self‐perception, which is likely, it seems natural that we experience ourselves as bipartite, comprising both a material and an immaterial dimension. As discussed above, because the brain and its neuronal mechanics are transparent, there is no contradiction in experiencing our mental dimension as coexisting with and being independent of the brain's material substrate. For the same reason, there is also no contradiction in assuming an autonomous agent, the self, which perceives, deliberates, decides, assigns meaning and acts. This assumption fulfils our need to identify causes for our actions, and our intuition readily accepts mental causation because our cultural embedding familiarized us with the notion that immaterial realities can be powerful agents.

When framed this way, a naturalistic explanation for the origin of the perceived dichotomy between mind and matter seems plausible, making the hard problem of consciousness appear less intimidating. Nevertheless, our intuition struggles to close the gap between the body—the result of biological evolution—and the self‐assigned immaterial dimension that has developed through cultural evolution. Yet this conflict may also have a naturalistic basis if one considers that the sources of our intuitions rely on prior dependent perceptions, which are viewed as truthful. Perceptions cannot be easily relativized or altered by rational arguments; visual illusions persist even when the observer understands the neuronal processes behind them. We must cope with this cognitive dissonance. Related views have been expressed by Sellars (1997) and specifically with respect to the mitigation of the ‘hard problem’ by Dennet (1991, 2019).

Discrepancies between intuition and rational insight are quite common and extend beyond just the mind–matter problem. They arise as an inevitable consequence of the natural sciences, which continuously reveal properties of nature beyond our intuitive grasp. This occurs because our intuitions rely on information from sensory systems evolved for survival in specific ecological niches, functioning in a highly idiosyncratic manner. Scientific discoveries frequently tear down the boundaries between phenomena that our sensory systems perceive as distinct. Consider the categories of space and time, matter and energy, or animals and humans. Although the unifying concepts derived from science may not alter our perceptions and intuitions, we seem to be able to adapt to these new science‐based ideas. Perhaps the hard problem will face a similar outcome. After all, the intuitive divide between mental phenomena and neuronal mechanisms is just one of many examples of the cognitive dissonances generated by the natural sciences.

## Conflicts of Interest

The author declares no conflicts of interest.

### Peer Review

The peer review history for this article is available at https://www.webofscience.com/api/gateway/wos/peer‐review/10.1111/ejn.70143.

## Data Availability

The author has nothing to report.
